# The First Case of Rowell Syndrome with Lupus Nephritis and Lobar Pneumonia in a Male Child Reported in Pakistan

**DOI:** 10.7759/cureus.4604

**Published:** 2019-05-07

**Authors:** Sumreen Shahid, Maria Khan, Laila Tul Qadar, Manahil Akmal, Ammarah Jamal

**Affiliations:** 1 Miscellaneous, Civil Hospital Karachi, Karachi, PAK; 2 Internal Medicine, Dow University of Health Sciences, Karachi, PAK; 3 Pediatrics, Civil Hospital Karachi, Karachi, PAK

**Keywords:** rowell syndrome, lupus erythematosus, systemic lupus erythematosus, lupus nephritis, lobar pneumonia, erythema multiforme

## Abstract

Rowell syndrome (RS) is a rare entity that presents with lupus erythematosus (LE), erythema multiforme (EM) like lesions and characteristic immunological findings including positive rheumatoid factor (RF), speckled antinuclear antibody (ANA) and positive anti-Ro or anti-La antibodies in the serum. Only a few cases have been reported in the literature, mostly in middle-aged women. Our case is about an 11-year-old male child who had a history of pustular rashes over the whole body with scattered targetoid lesions for the past few months and later developed respiratory distress and swelling of the body. He was extensively investigated and confirmed on specific positive immunochemistry markers as RS along with lobar pneumonia (LP) and lupus nephritis. The co-existence of lobar pneumonia in our patient was a unique feature that has not been previously reported.

## Introduction

Rowell syndrome (RS), first described in 1922 by Scholtz et al., was later characterized as a separate syndrome by Rowell and his colleagues in 1963 when they encountered four women who were suffering from systemic lupus erythematosus (SLE) and had developed rashes suggestive of erythema multiforme (EM) [1-2].

Since then 33 cases of RS have been reported in the literature, mostly affecting middle-aged women [3]. In 2008, Zeitouni et al. defined the major diagnostic criteria of RS consisting of pre-existing lupus erythematosus (LE) getting superimposed by EM and speckled pattern of antinuclear antibody (ANA), along with at least one minor criterion consisting of positive rheumatoid factor (RF), chilblains or the presence of anti-Ro or anti-La antibodies [4].

We report a rare case of RS at an unusual age of 11 years with an unfamiliar presentation of lobar pneumonia (LP).

## Case presentation

**Figure 1:**
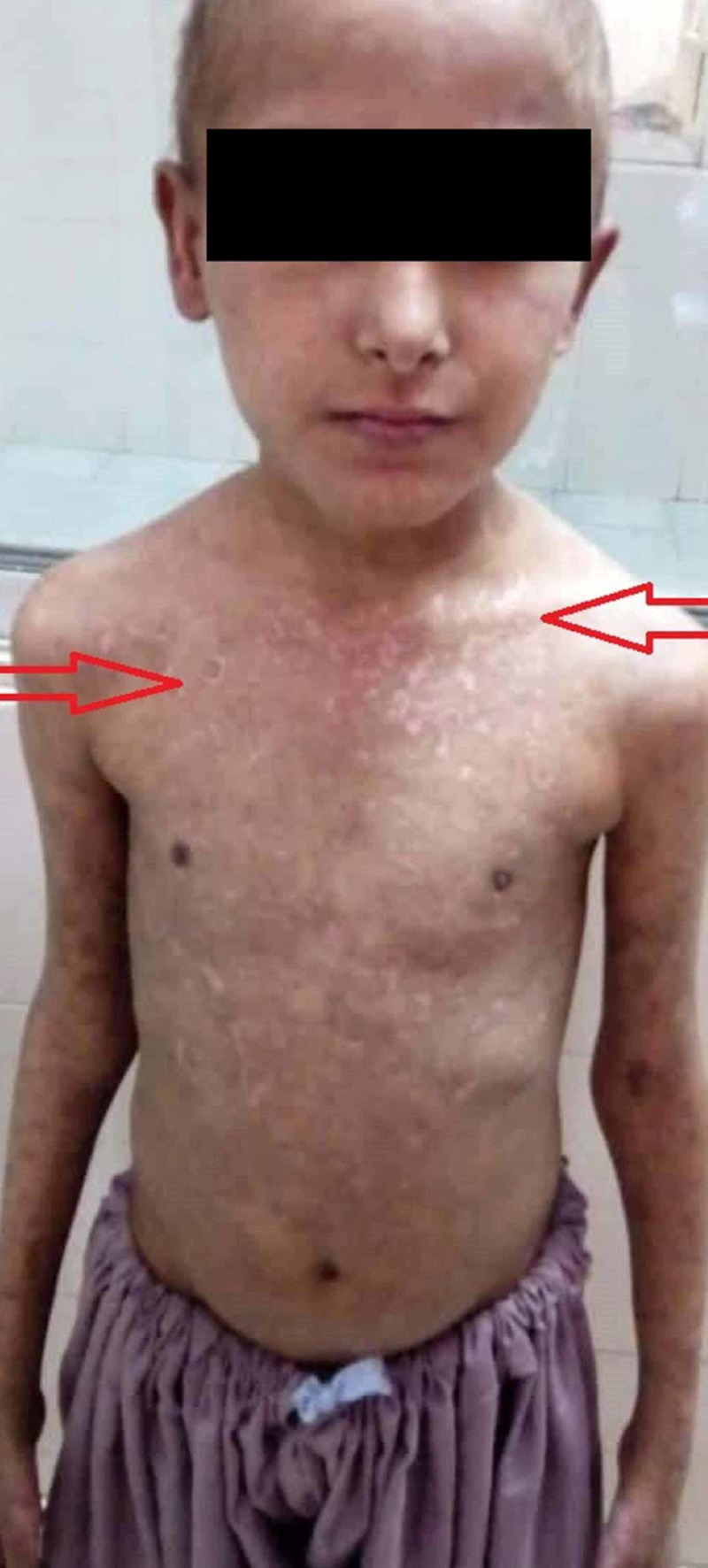
Scaly rash with occasional targetoid lesions all over the body

**Figure 2:**
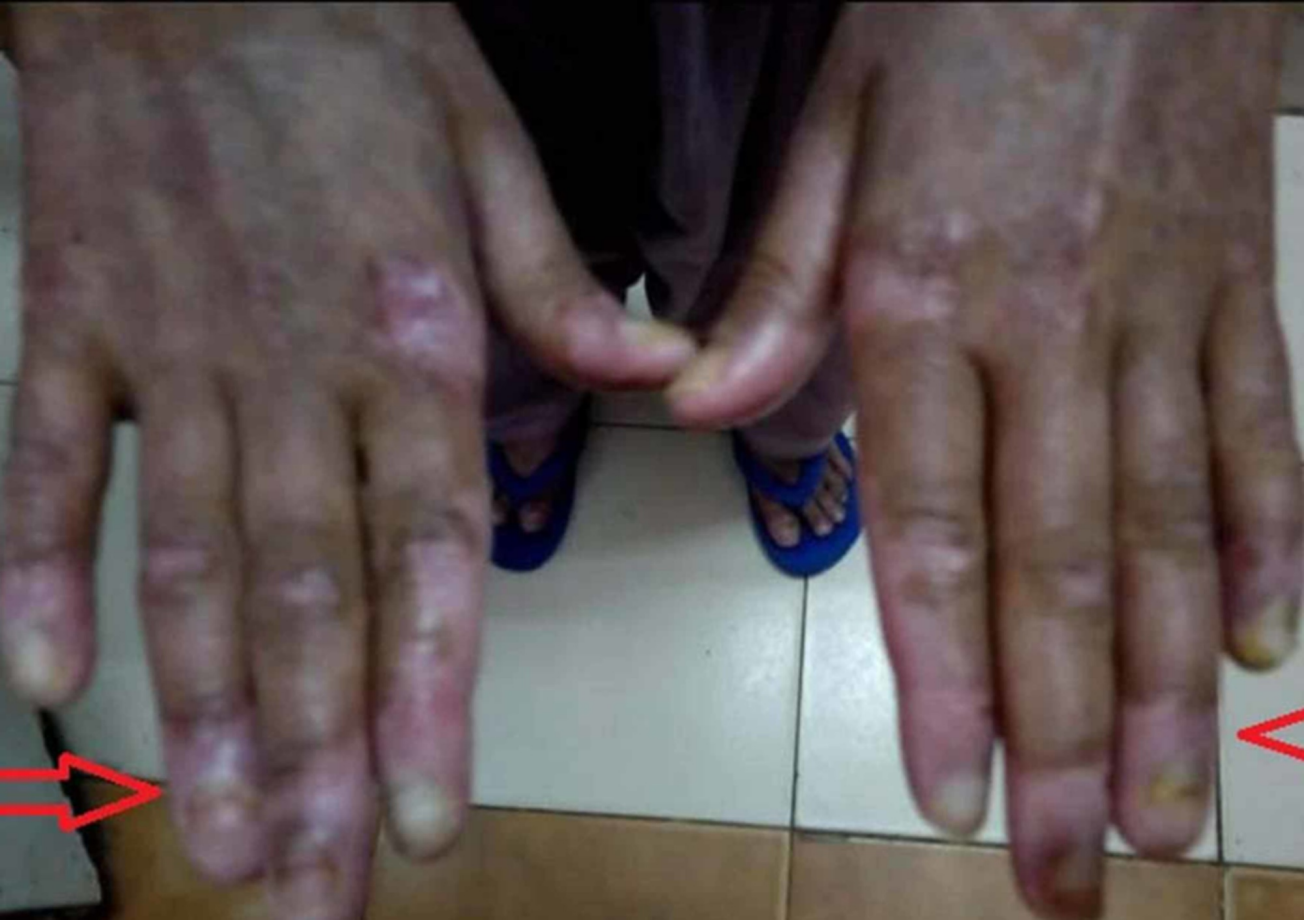
Loss of fingernails with peeling of skin

An 11-year-old, previously well, vaccinated male child was admitted in the pediatric ward of Dr. Ruth KM Pfau, Civil Hospital Karachi (CHK) with a two-day history of fever, cough, and abdominal distension followed by respiratory distress. His illness started seven months earlier with the eruption of pruritic, blistering rashes all over the body accompanied by a pussy discharge. At the time of onset, the rash was associated with low-grade fever, skin hyperpigmentation, oral ulcers and joint pains without any swelling or movement restriction. The rash usually appeared in successive generations on the sun-exposed regions progressing into a darkly pigmented scar over a period of one week. He consulted various doctors and was even hospitalized once for the rash but showed only temporary improvement to intravenous antibiotics and prednisolone. On examination, he showed obvious signs of respiratory distress with a respiratory rate of 40 breaths/min and a heart rate of 90 beats/min. Examination of the chest revealed signs consistent with left-sided LP. In addition, he had a scaly rash with occasional targetoid lesions all over the body associated with peeling of the skin (Figure 1), ruptured blisters, loss of fingernails (Figure 2) and sparse, brittle and depigmented hairs. He had hepatomegaly with a liver span of 15 cm and signs of free fluid in the abdomen.

**Figure 3:**
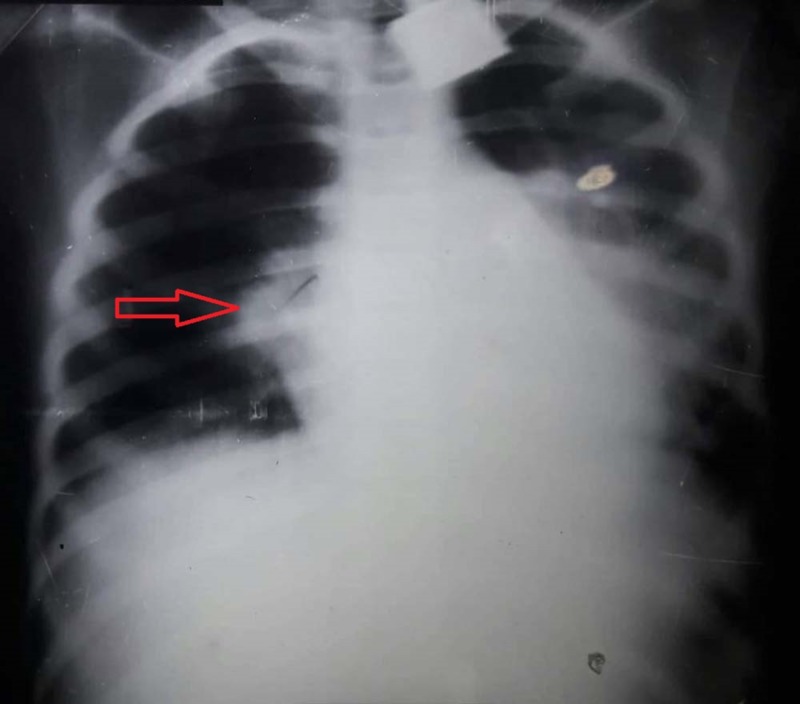
X-ray chest showing right-sided lobar pneumonia

The initial differential diagnosis for the skin rashes and nail dystrophy included SLE, mixed connective tissue disorders, hypohidrotic ectodermal dysplasia, and dyskeratosis congenita. On investigation, a full blood count showed moderate hypochromic, microcytic anaemia with haemoglobin of 8 g/dL. Raised erythrocyte sedimentation rate (ESR) of 98 mm/1st hr and raised C-reactive protein of 7.9 (<5 mg/L) were suggestive of inflammation. Serology revealed positive antinuclear antibody (ANA) and positive anti-double-stranded DNA (anti-ds DNA), both suggestive of SLE. Direct Coombs test was also positive. Urine DR showed protein ++, red cell casts and granular casts, which along with an increased ratio of spot urinary protein to creatinine of 4.5, suggesting glomerular involvement. Serum C3 and C4 levels were low, measuring 31 mg/dL (88-252 mg/dL) and 8 mg/dL (12-72 mg/dL), respectively, supporting the diagnosis of SLE. Renal and liver function tests were normal except for low serum albumin. Computed tomography (CT) scan of the abdomen confirmed hepatomegaly with marked ascites. X-ray chest showed right-sided LP (Figure 3). CT chest revealed bilateral multifocal multi-segmental ground glass haze along with multiple enlarged cervical, pretracheal and carinal lymph nodes, and bilateral mild pleural effusion, extending in the oblique fissure suggestive of active pulmonary infection.

Extractable nuclear antigen (ENA) panel revealed a negative anti-Scl-70, negative anti-Jo-1, a negative anti-La (SS-B), positive anti-ribonucleoprotien (RNP), positive anti-Smith (Sm) and a strongly positive anti-Ro (SS-A) antibodies, findings being consistent with the diagnosis of RS. We proceeded with the skin biopsy which revealed interface dermatitis showing lymphocytes and keratinocytes in the epidermis, supporting the diagnosis of erythema multiforme. A diagnosis of RS with lupus nephritis and LP was established based on the clinical manifestations and positive immune-chemistry. The patient was treated with methylprednisolone initially followed by oral prednisolone, azathioprine, naproxen and hydroxychloroquine on the advice of pediatric rheumatologist and antibiotics for pulmonary infection. His rashes improved and proteinuria resolved over a period of two weeks. The patient is under regular follow-up with no recurrence of skin lesions or pneumonia.

## Discussion

Rowell syndrome is a rare disorder characterized by the coexistence of LE with EM. The association was first reported by Scholtz [1]. In 1963, Rowell, Beck and Anderson described 4 female patients who had a similar association. Immunological findings of these patients included a speckled pattern of ANA, positive RF, and the anti-SjT type, which is now considered similar to anti-Ro antibody [2].

Our case meets all the diagnostic criteria of RS, both major and minor making it the first well-defined case of RS from Pakistan. It is important to mention that most of the cases of RS have been reported in middle-aged women; hence the frequency of RS in children is quite rare. A similar case was reported in a 15-year-old boy from India; however, our case presented with a unique association of respiratory infection coexistent with the RS which, to our knowledge, has not been reported in the literature so far [5]. These clinical findings, although not a part of RS, could emerge as important associations of the RS in future as more data on RS become available; however, in the present case, this appears to be an incidental finding rather than the consistent feature of the syndrome.

The therapeutic regimen used for RS is similar to that of SLE. Majority of the reported cases showed satisfactory response to corticosteroids with azathioprine or antimalarial drugs, such as chloroquine or hydroxychloroquine [4-6]. Our patient responded well to azathioprine, prednisolone, hydroxychloroquine, and naproxen. A similar case reported another successful treatment option with low-dose cyclosporin A, adding another alternative treatment option for patients refractory to standard therapies [7].

Our literature search also revealed several debates concerning whether RS should be considered as a unique clinical case. Approximately 88% of the cases report speckled ANA pattern as the most important feature of RS; however, this is shared often with SLE, mixed connective tissue disease and scleroderma. Moreover, the presence of anti-Ro/La antibodies can also be detected in other disorders such as subacute cutaneous lupus erythematosus (SCLE), Sjögren syndrome, SLE, rheumatoid arthritis, and scleroderma [7-9].

## Conclusions

The above-mentioned case adds to the existing scientific literature on RS and highlights the need to consider RS as a differential diagnosis in all patients with LE with EM-like lesions and also the possibility of its occurrence in children in addition to adults. The co-existence of LP in our patient was a novel finding in the setting of RS. Our patient responded well to the conventional treatment strategies employed for RS, indicating the adequacy of the currently accepted therapeutic regimens; however, thorough research needs to be undertaken to establish the existence of RS as a separate entity.
